# Pleiotropic tumor suppressor functions of WWOX antagonize metastasis

**DOI:** 10.1038/s41392-020-0136-8

**Published:** 2020-04-17

**Authors:** Saleh Khawaled, Giovanni Nigita, Rosario Distefano, Sara Oster, Sung-Suk Suh, Yoav Smith, Abed Khalaileh, Yong Peng, Carlo M. Croce, Tamar Geiger, Victoria L. Seewaldt, Rami I. Aqeilan

**Affiliations:** 1 Lautenberg Center for Immunology and Cancer Research, Hebrew University-Hadassah Medical School, IMRIC, Jerusalem, Israel; 2 Department of Cancer Biology and Genetics, Wexner Medical Center, The Ohio State University, Columbus, OH USA; 3 Department of Bioscience, Mokpo National University, Muan, Republic of Korea; 4 Genomic Data Analysis Unit, Hebrew University-Hadassah Medical School, Jerusalem, Israel; 5 Department of Surgery, Hadassah Medical Center, Jerusalem, Israel; 6 Department of Thoracic Surgery, State Key Laboratory of Biotherapy, West China Hospital, Sichuan University and Collaborative Innovation Center for Biotherapy, 610041 Chengdu, China; 7 Department of Human Molecular Genetics and Biochemistry, Sackler Faculty of Medicine, Tel Aviv University, Tel Aviv Israel; 8 Department of Population Sciences, City of Hope Comprehensive Cancer Center, Duarte, CA USA

**Keywords:** Genetics research, Metastasis

## Abstract

Tumor progression and metastasis are the major causes of death among cancer associated mortality. Metastatic cells acquire features of migration and invasion and usually undergo epithelia-mesenchymal transition (EMT). Acquirement of these various hallmarks rely on different cellular pathways, including TGF-β and Wnt signaling. Recently, we reported that WW domain-containing oxidoreductase (*WWOX*) acts as a tumor suppressor and has anti-metastatic activities involving regulation of several key microRNAs (miRNAs) in triple-negative breast cancer (TNBC). Here, we report that WWOX restoration in highly metastatic MDA-MB435S cancer cells alters mRNA expression profiles; further, WWOX interacts with various proteins to exert its tumor suppressor function. Careful alignment and analysis of gene and miRNA expression in these cells revealed profound changes in cellular pathways mediating adhesion, invasion and motility. We further demonstrate that WWOX, through regulation of miR-146a levels, regulates SMAD3, which is a member of the TGF-β signaling pathway. Moreover, proteomic analysis of WWOX partners revealed regulation of the Wnt-signaling activation through physical interaction with Disheveled. Altogether, these findings underscore a significant role for WWOX in antagonizing metastasis, further highlighting its role and therapeutic potential in suppressing tumor progression.

## Introduction

Metastasis is responsible for as much as 90% of cancer associated mortality. During metastasis, a cancer cell from the primary tumor invades the surrounding tissue of the tumor, enters into the bloodstream (intravasation), survives as it moves through the blood stream into distant tissues, exits the bloodstream (extravasation), survives the microenvironment of the distant tissue and finally colonizes in a new microenvironment where it begins to proliferate.^1,2^ Cells that leave the primary tumor and initiate the metastatic process must acquire the capabilities of migration and invasion; cells usually acquire these mesenchymal traits as a consequence of epithelial-to-mesenchymal transition (EMT). In general, the metastatic cascade can be divided into two main stages: physical translocation of a cancer cell from the primary tumor to a distant site in a distant tissue and colonization of the cells at the distant site.^3^ The latter step depends on the ability of the cancer cells to revert back to an epithelial state as a consequence of the mesenchymal-to-epithelial (MET) process. Several genes and circuits have been proposed to regulate EMT, MET and metastasis, among which are the Wnt and TGFβ pathways.

The Wnt signaling pathway is involved in many processes that are essential for development, adult homeostasis and tumorigenesis.^4,5^ This signaling can be divided into canonical and non-canonical pathways. The canonical pathway requires Wnt ligand binding to the Frizzled receptors as well as to the LRP5/6 co-receptors to initiate intracellular signaling via β-catenin nuclear translocation. Wnt ligand binding to Frizzled receptors in association with LRP5/6 induces dishevelled (DVL) phosphorylation, which subsequently recruits Axin, thereby deconstructing the degradation complex and achieving β-catenin stabilization and subsequent nuclear translocation.^6–9^ The non-canonical pathway, which is often referred to as the β-catenin-independent pathway, is subclassified into two kinds: the planar cell polarity pathway, which involves jun N-terminal kinase (JNK), and the Wnt/Ca2+ pathway. Several lines of evidence have associated Wnt signaling with EMT and metastasis.^10–12^ Recently, by combining a screen of human breast cancer samples with meta-analysis of previous breast cancer microarray studies, it has been shown that the Wnt pathway is more active in TNBC subtypes than it is in other subtypes. Moreover, increased Wnt/β-catenin signaling is associated with tumors of high grade and poor prognosis and with metastatic disease, especially in the brain and the lungs.^13^

The TGFβ pathway signals through well characterized transmembrane serine-threonine kinase receptors and intracellular signaling molecules of the SMAD family. Their biological effects are context dependent, and they vary based on tissue type and the activity of other intracellular signaling pathways in the cell. They are potent negative growth regulators that can induce differentiation, apoptosis, cell migration, adhesion, and extracellular matrix deposition. Although TGFβ is a strong growth inhibitor, elevated TGFβ signaling in tumors contributes to carcinoma progression and metastasis.^14^ In many types of cells, TGFβ-induced growth inhibition is mediated through SMAD-dependent inhibition of the expression of the c-MYC oncogene, cyclin-dependent kinases (CDKs) and CDC25A.^15,16^ It is poorly understood how TGFβ switches from a tumor suppressor to a promoter of tumorigenesis and metastasis. One study suggested that this switch occurs due to other proteins, such as 14-3-3ζ.^17^ In a different study, it was shown that TGFβ, which is produced abundantly by stromal cells, stimulates the expression of ANGPTL4, which itself disrupts the capillary vascular endothelial cell junctions and enables dissemination of breast cancer cells to initiate lung metastasis.^18^ Identifying novel regulators of Wnt and TGFβ signaling pathways would greatly enhance our understanding and management of metastasis.

The WW domain-containing oxidoreductase (*WWOX*) gene spans a genomic locus of more than 1 Mbp; the gene contains nine exons and encodes an open reading frame of 1245 bp. The gene spans the fragile site FRA16D and includes a genomic region involved in chromosome translocation in multiple myelomas and hemi- and homozygous deletions (HDs) in cancers. WWOX protein expression, by means of immunohistochemistry and Western blot, was shown to be reduced or lost in many malignancies,^19–21^ and it has been associated with clinical or pathological parameters^20,22–26^ and with poor prognosis.^27–29^ In a comprehensive study that included 3131 cancer specimens, a high-resolution analysis of somatic copy-number alterations revealed that deletion of *WWOX* is a common event in human cancer.^30^ Modeling loss of WWOX expression in murine mammary gland epithelium resulted in increased incidence of TNBC-like and basal-like breast cancer (BLBC) tumors.^31–33^ Indeed, both low copy number and reduced mRNA expression of WWOX are associated with advanced stages of TNBC, implying that WWOX plays a significant role in TNBC progression.^34^ WWOX mediates many of its functions through its ability to interact with other proteins via its WW1 domain.^35–37^ Our recent observations demonstrated that WWOX can regulate the levels of several miRNAs, which regulates TNBC metastasis.^34^ In particular, we showed that WWOX could regulate the c-MYC/miR-146a/Fibronectin axis to antagonize TNBC invasion and tumor growth.^33,34^ In other reports, it has been proposed that WWOX loss promotes metastasis of TNBC cells through regulating the JAK2/STAT3 axis,^38,39^ further emphasizing the role of WWOX in antagonizing tumorigenesis. Moreover, a recent study showed that WWOX-deficient metastatic cells actively evade WWOX positive cells in their environment and then utilize various signaling pathways in order to force WWOX positive cells to undergo apoptosis, allowing further progression of metastasis.^40^ These observations and others prompted us to further investigate possible roles of the tumor suppressor WWOX in cancer progression and metastasis.

In this study, we aimed to identify comprehensive molecular and proteomic changes in WWOX-expressing cancer cell lines. By combining mRNAs and miRNAs data analysis we found that adhesion, motility and invasion promoting cellular pathways are downregulated upon WWOX restoration. Of particular interest, both Wnt and TGF-β signaling pathways were significantly enriched. Moreover, we validated that miR-146a also targets SMAD3, an activator of TGF-β signaling. By using proteomics, we demonstrated that WWOX specifically interacts with several proteins, some of which were novel and some that were known. We confirmed that WWOX interacts with DVL2 and demonstrated that it negatively regulates Wnt/b-catenin signaling in TNBC cells. These findings further imply that WWOX performs a plethora of tumor suppressor functions, including antagonizing cancer development and progression.

## Results

### WWOX restoration in highly metastatic cancer cells alters mRNA expression

**Fig. 1:**
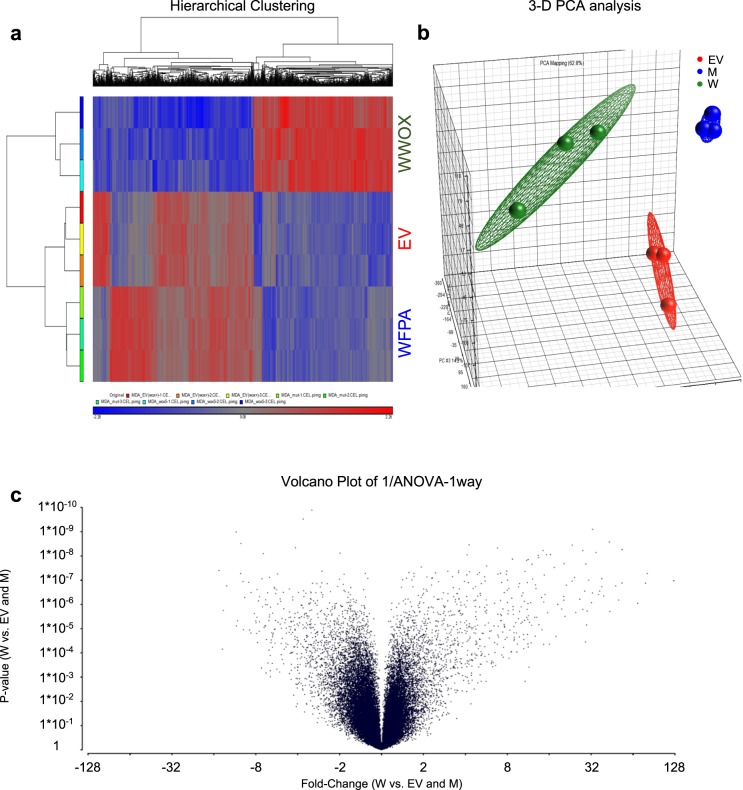
mRNAs profiling using Affymetrix analysis. **a** Hierarchical unsupervised clustering of gene expression in MDA-MB435S cells revealed the presence of four major clusters. Of these, two clusters included genes that were strongly upregulated or downregulated in WWOX (blue) cells compared to EV (red) or WFPA (green) cells. **b** PCA results show the clustering of the replicates from each clone, emphasizing the clustering of the EV (RED) and WFPA (blue) in one dimension that is separate from that of WWOX (green). **c** Volcano plot analysis showing upregulated and downregulated genes comparing WWOX-expressing cells to EV and WFPA-expressing cells

Our previous work demonstrated that changes to WWOX are common in several cancers, and such changes are correlated with poor prognosis, including in BLBC and TNBC.^31,33,34^ To further analyze the effect of WWOX on metastasis formation, we studied the differential expression of mRNAs using an Affymetrix GeneChip in WWOX-expressing and deficient metastatic cells. To this end, we performed mRNA profiling of MDA-MB435S metastatic melanoma cells expressing an empty-vector (EV), WWOX and a WWOX-WFPA mutant (Supplemental Fig. 1). The latter mutation has been shown to disrupt WWOX interaction with other proteins and abrogate the tumor suppressor activity of WWOX.^35,41^ The analysis revealed numerous changes in gene expression in WWOX-expressing cells when compared to the control and mutant cells (Supplementary Table 1). Indeed, hierarchical unsupervised clustering of the differentially expressed genes revealed two major clusters (Fig. 1a). A three-dimensional principal component analysis (PCA) confirmed this clustering, clearly showing that EV (red) and WFPA (blue) cells clustered together apart from the WWOX (green) cells (Fig. 1b). When analyzing the differentially expressed genes we found 833 downregulated genes and 724 upregulated genes (Fig. 1c, Supplemental Table 1). We then further examined these differentially expressed genes by performing pathway enrichment analysis. Using the Enricher Website (http://amp.pharm.mssm.edu/Enrichr/) and the KEGG database, we found cell adhesion, motility, and invasion pathways to be among the top pathways that were enriched (Supplementary Tables 2, 3). Interestingly, the JAK2/STAT3 signaling pathway, that has been recently associated with WWOX in TNBC^38^ appeared as one of the most enriched pathways in the downregulated gene set (Supplementary Table 2). These results further confirm that WWOX expression leads to significant transcriptomic changes that are associated with cellular phenotypes that antagonize metastasis.

### mRNA-miRNA expression comparison reveals WWOX regulated pathways

**Fig. 2:**
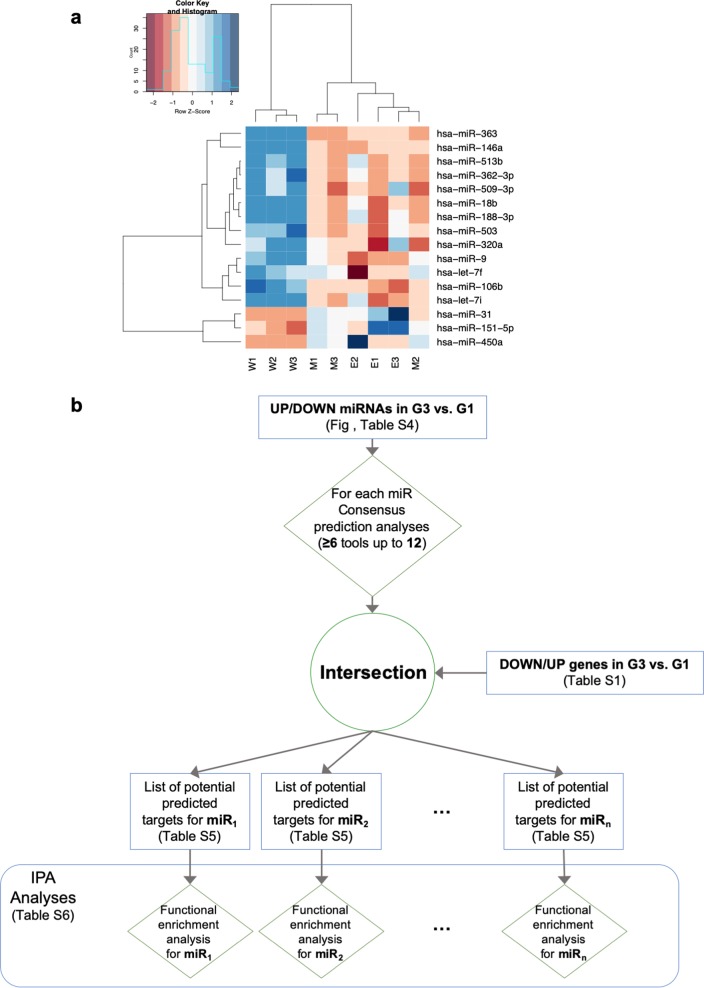
miRNAs profiling in WWOX-expressing MDA-MB435S metastatic cells and workflow of the intersection analysis with mRNA profiling. **a** Heatmap with candidate miRNAs. “W” samples are wild type, “E” samples are empty vector and M samples are mutated WWOX ones. **b** Workflow of the miRNA targeting prediction and function prediction analyses

**Fig. 3:**
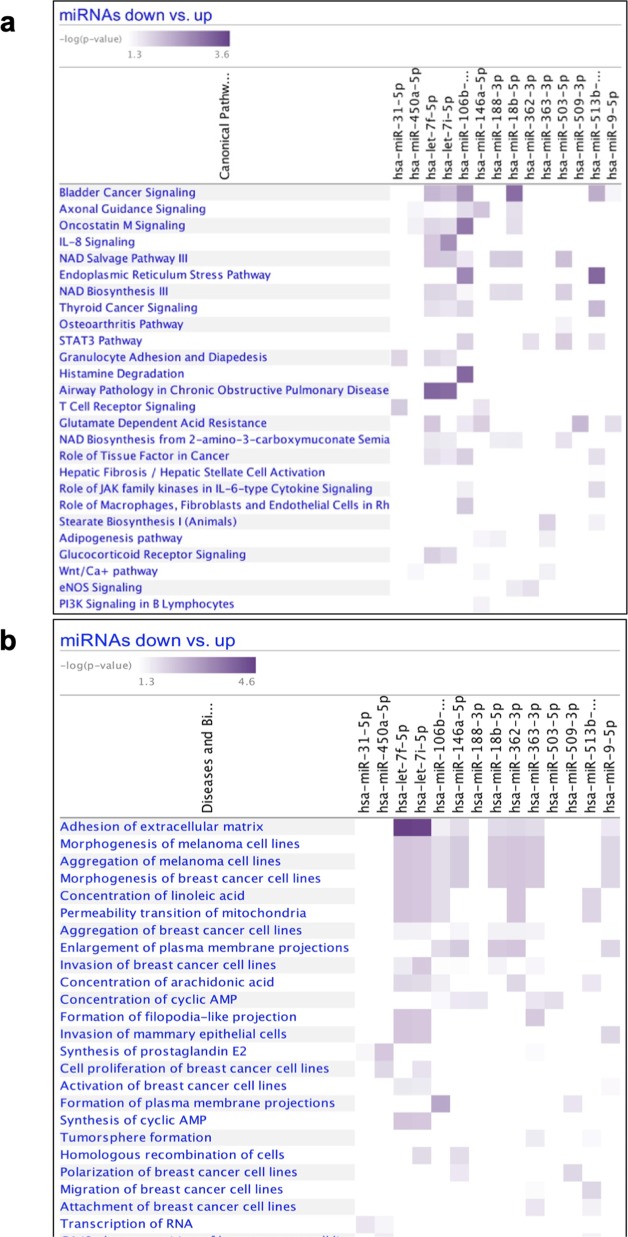
Outcome of mRNA-miRNA intersection analysis. **a** Pathway enrichment analysis on the validated miRNA targets. Heat map showing pathway enrichment analysis for the miRNA targets that were changed upon WWOX expression (both up- and downregulated). Scale-bar shows the −log(*P*-value); 3.6 is the lowest *P*-value (=0.000251). **b** Diseases and biological function analysis on the validated targets of miRNAs. Heat map showing diseases and biological function enrichment analysis for the targets of miRNAs that were changed upon introduction of WWOX expression (both up- and downregulated). Scale-bar shows the -log(P-value); 1.3 = 0.05 *P*-value

Our previous findings revealed that WWOX promoted the expression of several miRNAs to antagonize cell invasion and metastasis,^34^ which is similar to what we found when analyzing the mRNA profiling. These common effects of WWOX led us to consider whether comparing expression results found in mRNA (from this study) and miRNA^34^ (Fig. 2a) datasets could result in further enrichment of WWOX regulated pathways. To address this, we compared the lists of mRNAs (Supplementary Table 1) and the predicted targets from the miRNAs GeneChip experiments (Supplementary Table 4) that were performed on the same MDA-MB435S cells (see Methodology section and Fig. 2b). We found that several predicted targets of the downregulated miRNAs, as shown by Nanostring microarrays, were upregulated in the Affymetrix mRNA analysis, and vice versa (Supplemental Table 5). After this phase, for each miRNA we performed a pathway enrichment analysis for the “strong predicted” targets (see Methodology section) using Ingenuity Canonical Pathways software. We found that most of the pathways that were significantly enriched are connected to cellular adhesion and invasion such as axonal guidance signaling, STAT3 pathway and Wnt/Ca+ pathway (Fig. 3, Supplementary Table 6), further underscoring the function of WWOX in antagonizing hallmarks of metastasis.

### miR-146a targets TGFβ signaling pathway

**Fig. 4:**
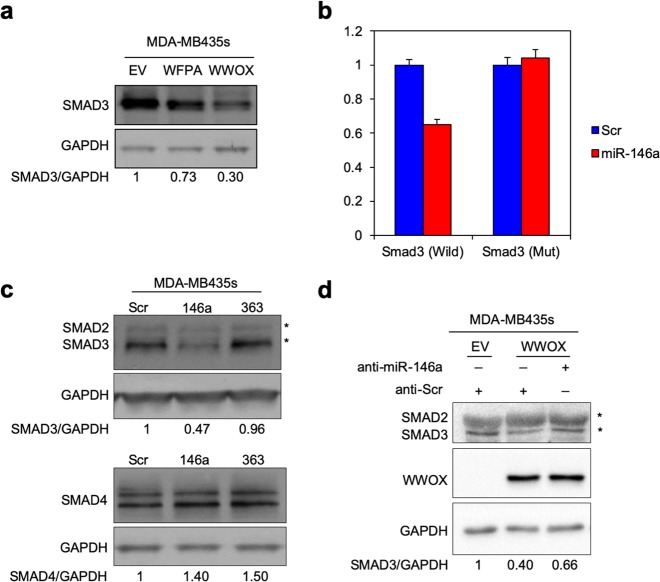
WWOX modulates TGF-β signaling via miR-146a targeting of SMAD3. **a** Immunoblot of SMAD3 in MDA-MB435 cells; Ev-empty vector, WFPA-expressing mutated WWOX, WWOX-expressing wild-type WWOX. **b** Luciferase assay of *SMAD3* 3′UTR (wild-wild type, mut-mutated 3′UTR), with or without miR-146a. Bars indicates SD. **c** Immunoblot showing SMAD3 and SMAD2 levels in MDA-MB435S cells upon expression of miR-146a or miR-363. **d** Immunoblot showing Smad3 in MDA-MB435S cells; Ev-empty vector, and WWOX-expressing wild-type WWOX, and assays were performed after expression of anti-miR-146a or anti-Scr for 24 and 48 h. GAPDH was used as an endogenous control in (**a**), (**c**) and (**d**). Densitometry analysis for SMAD3 levels is shown below each blot

We previously demonstrated that WWOX enhances the expression of miR-146a, which targets Fibronectin (FN1) and leads to reduced invasion and metastasis.^34^ This observation and our current analysis (Figs. 2, 3) led us to question whether effects of WWOX as an anti-metastatic modulator are mediated through targeting other effectors. One prominent way that WWOX exerts its tumor suppressor activity is through inhibition of the TGFβ pathway.^42,43^ Another study has shown that protein–protein interaction between WWOX, through its WW1 domain, and the PPGY motif of SMAD3, leads to the inhibition of SMAD3 transcriptional activity.^44^ Moreover, the TGFβ pathway was among the significant altered pathways upon introducing WWOX expression in MDA-MB435S cells (Supplementary Table 2). Therefore, we aimed to determine whether miR-146a, as a downstream effector of WWOX, might also target the TGFβ signaling pathway, particularly SMAD3, and hence affect metastasis. Remarkably, we found that SMAD3, a main effector of the TGFβ activation, was reduced upon the introduction of WWOX expression, but the WFPA mutant had much less effect (Fig. 4a). Intriguingly, we found that miR-146a directly targets SMAD3, as evident by the repression of expression from the wild-type 3′UTR of SMAD3. This repression was abolished upon mutating the 3′UTR (Fig. 4b). Moreover, expression of miR-146a, led to reduced levels of SMAD3, but not of SMAD4, another known binding partner of WWOX; expression of miR-363, which is not predicted to target SMAD3, had no effect on SMAD3 levels^45^ (Fig. 4c). Rescue experiment using anti-miR-146a resulted in the increase of SMAD3 expression in WWOX-expressing cells, evident at 24 h after transfection (Fig. 4d). This observation, along with previously described mechanisms, highly suggests that WWOX inhibition of metastasis could also affect the TGFβ signaling pathway.

### The WWOX interactome in highly metastatic cells reveals that Wnt signaling is a major pathway

**Fig. 5:**
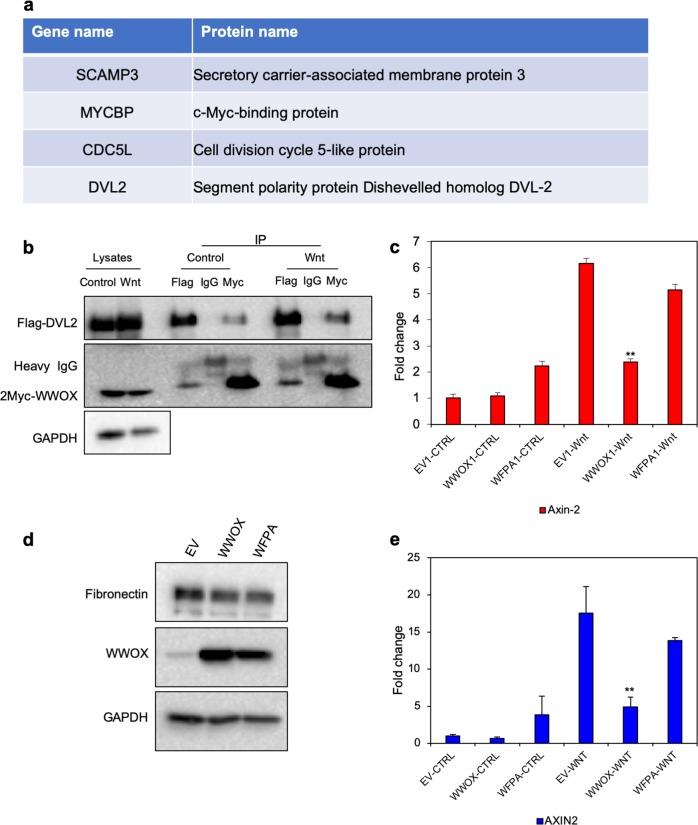
WWOX physically interacts with DVL2 and functionally results in reduced Wnt pathway activation. **a** WWOX-interacting proteins in MDA-MB435S cells. **b** Immunoblot showing the co-IP of WWOX and DVL2 from HEK293T cells using Flag tagged DVL2 and Myc-tagged WWOX; mouse IgG was used as a negative control. Control- without Wnt ligands, Wnt- with Wnt ligands. Heavy chain IgG is indicated for each lane to demonstrate equal amounts of precipitate. **c** qRT-PCR of *AXIN2* in MDA-MB231 cells treated with or without Wnt ligands; *UBC* was used as an endogenous control. The number of times an experiment was repeated (N) is stated in the legend. **d** Immunoblot showing Fibronectin and WWOX in SUM149 cells; EV- empty vector, WWOX- wild-type WWOX, and WFPA- mutated WWOX. **e** qRT-PCR of *AXIN2* in SUM149 cells treated with or without Wnt ligands; *UBC* was used as an endogenous control. *GAPDH* was used as an endogenous control in (**a**) and (**c**). **P-value < 0.01. Statistical analyses (including error bars and *p* values) for (**c**) and (**e**) represent three independent experiments

WWOX is known as a scaffold protein due to its WW domains and protein–protein interaction ability. As described earlier, disruption of the first WW domain (through point mutations such as the WFPA), leads to disrupted interactions with partner proteins.^35,41^ To assess the interactome map of WWOX in our system, we attempted to identify the WWOX interaction proteome using mass spectrometry (MS). To this end, immunoprecipitations (IPs) were performed using a monoclonal anti-WWOX antibody on lysates from MDA-MB435S control cells, MDA-MB435S cells overexpressing WWOX or those overexpressing WWOX-WFPA. Precipitates were washed, eluted and assessed by MS. After analyzing the MS data (Supplementary Table 7), we searched for the proteins that were enriched in the WWOX group and not in the EV and the WFPA groups. We identified four proteins (MYCBP, DVL2, SCAMP3, and CDC5L) that were specific to the WWOX group (Fig. 5a). WWOX was previously reported to bind to DVL, which is a member of the Wnt/β-catenin pathway.^35,46^ The Dvl proteins play a key role in three different pathways activated upon Wnt receptor activation: the canonical Wnt/β-catenin pathway, the planar cell polarity pathway and the Wnt/Ca2+ pathway.^47^ Wnt signaling has well established roles in human breast cancer, as elevated levels of nuclear and/or cytoplasmic β-catenin are detectable by immunohistochemical staining in a majority of breast tumor tissue samples.^48,49^ Physical interaction between WWOX and DVL2 leads to the inhibition of the Wnt/β-catenin pathway, suggesting one additional mechanism by which WWOX executes its tumor suppressor function.^46^ To verify these findings in our system, we validated WWOX-DVL2 interaction by exogenously expressing the two proteins in HEK293T in the presence or absence of Wnt ligands. Following transfection, we performed co-immunoprecipitation (co-IP) assays, using Flag or Myc antibodies, and then we performed immunoblotting experiments. As seen in Fig. 5b, physical interaction between WWOX and DVL2 was evident even without Wnt ligands. Addition of Wnt ligands to the cells further enhanced the binding between WWOX and DVL2 (Fig. 5b). Additionally, nuclear β-catenin levels were reduced when WWOX was ectopically expressed in MDA-MB231 metastatic breast cancer cells (Supplemental Fig. 2). To further explore the outcome of WWOX-DVL2 interaction, we examined the effect of WWOX expression on Wnt pathway activation by assessing levels of AXIN2, which is a direct target of Wnt pathway activation. Restoration of WWOX but not WWOX-WFPA in MDA-MB231 cells led to reduced mRNA levels of *AXIN2*, as assessed by qRT-PCR (Fig. 5c). Similar results were observed in SUM149 metastatic breast cancer cells (Fig. 5d, e). Consistent with our previous findings,^34^ reduction of Fibronectin upon restoration of WWOX was also evident in SUM149 cells (Fig. 5d). These results imply that WWOX negatively regulates the canonical Wnt signaling pathway, providing another mechanism by which it antagonizes tumorigenesis and metastasis.

## Discussion

WWOX is a tumor suppressor whose functions extend to antagonizing cell growth and metastasis.^34,38,50^ Our current comprehensive analysis suggests that WWOX expression suppresses tumorigenesis through pleotropic functions that are exerted at mRNA, miRNA and protein levels. We present evidence that introduction of WWOX expression in highly metastatic cancer cells affects several gene hubs and signaling pathways that are linked to migration, invasion and metastasis.

Our observations strongly suggest that WWOX is a general regulator of transcription and post-transcriptional regulation. This prompted us to explore the significant and recurrent changes of mRNA and miRNA expression in WWOX-expressing cells. Indeed, we found that many of the transcripts that were changed upon introducing WWOX expression are also targets of the miRNAs that were changed upon WWOX overexpression (downregulated mRNAs matched targets of upregulated miRNAs and vice versa). In concordance with our previous pathway enrichment analysis, analysis of all the targets of the miRNAs that were upregulated identified pathways related to cell adhesion, motility and invasion; these results suggest that the net effect of WWOX restored expression is inhibition of invasion and metastasis. This effect of WWOX again is achieved through its actions at multiple cellular levels, leading to net inhibition of invasion and metastasis.

Previous work in our lab revealed intimate crosstalk between WWOX and proto-oncogene c-Myc in TNBC cells^34^; WWOX negatively regulates c-Myc function. How does WWOX affect c-Myc function? WWOX could bind c-Myc on the chromatin and modulate its regulation of target genes; one such target we demonstrated to be miR-146a. In fact, it has been previously shown that c-Myc represses miR-146a, leading to its downregulation.^51^ Hence, when WWOX is expressed, c-Myc repression of miR-146a is decreased, leading to upregulation of miR-146a. Another possibility is that WWOX might be interacting with and inhibiting specific proteins mediating co-activation of c-Myc. One piece of evidence for this idea stems from our current findings showing that WWOX might interact with MYCBP, c-Myc binding protein. MYCBP is especially known to bind the N-terminal region of c-Myc, corresponding to the transactivation domain, via its C-terminal region, which stimulates the activation of E box-dependent transcription of c-Myc.^52^ When WWOX is expressed, MYCBP might be sequestered, leaving c-Myc less active. Further investigation of the outcome of WWOX-MYCBP interaction is necessary to better understand WWOX inhibitory effect over c-Myc.

Restoration of WWOX enhances miR-146a expression in several TNBC and non-TNBC cell lines (like MDA-MB435S), leading to targeting of FN1 and altering cellular fate,^34^ i.e., EMT. Nevertheless, modulating levels of miR-146a could have far reaching effects on other venues and cellular pathways. We demonstrate in our study that miR-146a targets SMAD3, a known partner of WWOX^44^; however, it does not target SMAD4, which is another known binding partner of WWOX.^45^ This result is consistent with the current knowledge regarding the biological functions of these interactions. While the relationship between WWOX and SMAD4 is synergistic, leading to increased apoptosis and tumor suppression, the physical interaction between WWOX and SMAD3 is antagonistic and has been shown to result in the later cytoplasmic sequestration of SMAD3 and reduced transcriptional activity.^44^ Our current findings show that this inhibitory effect extends further than was previously known, by showing that miR-146a, an effector miRNA of WWOX, targets SMAD3 thus contributing to its anti-metastatic function. This observation further confirms the pleotropic tumor suppressor effects of WWOX antagonize tumor development and metastasis.

Additional pathways that are known to be involved in metastasis were altered by WWOX restoration. One such pathway is the Wnt signaling pathway. A number of WWOX effector miRNAs are shown to target the Wnt signaling pathway (Fig. 3). Furthermore, we found that WWOX physically interacts with DVL2 through protein–protein interaction (Fig. 5). DVL2 is a member of the DVL family of proteins. In canonical Wnt signaling, binding of Wnt to the Frizzled receptor helps recruit DVL, which thereby prevents constitutive degradation of β-catenin (reviewed in^53^). Furthermore, we found that this physical interaction reduces the functional outcome of Wnt signaling in TNBC cells, as shown by reduced *AXIN2* levels. Altogether, it is no surprise that the effect of WWOX on miRNA expression is also affecting Wnt signaling. These observations, in combination with other lines of support presented earlier, establish a complex scenario in which WWOX affects a variety of cellular pathways, contributing to a drastic shift in cell behavior toward an epithelial state, hence, leading to inhibition of metastasis.

Two additional proteins were identified as putative WWOX-interacting partners in MDA-MB435 cells, SCAMP3 and CDC5L. SCAMP3 is an isoform in the secretory carrier membrane protein (SCAMP) family. It is a membrane-trafficking protein involved in endosome transport. SCAMP3 was shown to be a marker associated with poor prognosis in hepatocellular carcinoma,^54^ and more importantly, it was shown to be upregulated in inflammatory breast cancer (IBC), which is a lethal form of breast cancer.^55^ Whether WWOX loss promotes SCAMP3 function in cancer cells is to be determined in future studies. In fact, another study has shown via MS that WWOX and SCAMP3 co-immunoprecipitate, further emphasizing a possible functional role for this interaction.^56^ CDC5L, cell division cycle 5-like, is a pre-mRNA splicing factor that is known to regulate mitotic progression. One study reported that depletion of CDC5L causes dramatic mitotic arrest, chromosome misalignment and sustained activation of the spindle assembly checkpoint, which eventually leads to mitotic catastrophe. Given that WWOX has been previously associated with DNA damage checkpoint proteins, such as ATM, ATR and p53 signaling, future studies should examine the functional association between WWOX and CDC5L on cell cycle control and the DNA damage response. Our MS analysis further reveal multiple interactions that are unique to WWOX-WFPA suggesting that these interactions are mediated through the SDR domain. Future in-depth analysis shall uncover the significance of these interactions.

### Concluding remarks

Overall, our work reveals that the gene product of FRA16D, which has been proposed to be altered at early stages of carcinogenesis (reviewed in refs. ^57,58^), might be affecting later steps of cancer progression and metastasis. One debatable question in the field is whether the ability to metastasize develops in tumors early on upon their formation or if it is acquired along the way.^59^ Whether loss of WWOX feeds into earlier seeding of metastatic cancer cells is to be determined. Our observations further argue against a passenger role of WWOX in tumorigenesis and suggest that its deletion/inactivation could generate a selective pressure that promotes tumorigenesis and progression. We argue that WWOX acts as an inhibitor of metastasis through changes in multicellular signaling pathways regulating cell adhesion, motility and survival that are regulated at multiple cellular and molecular levels. We conclude that therapeutic introduction of WWOX expression in early and late stages of carcinogenesis could have beneficial outcomes for cancer intervention.

## Materials and methods

### Cell culture and plasmids

MDA-MB435S (metastatic melanoma cancer) and MDA-MB231 (metastatic breast cancer) cells were grown in DMEM (Gibco) supplemented with 10% FBS, glutamine and penicillin/streptomycin (Biological Industries, Beit-Haemek, Israel). SUM149 (metastatic breast cancer) and HEK293 cells were grown in RPMI1640 (GIBCO) supplemented with 10% FBS, glutamine and penicillin/streptomycin. All cells were grown at 37 °C with 5% CO_2_. Cells were routinely validated and confirmed as mycoplasma-free, and cells from early passages were used. Stable clones of cells overexpressing WWOX were produced using a lentiviral vector (pDEST12.2^TM^ destination vector, Gateway Cloning Technology) containing either WT or mutated (WFPA) WWOX. Clones were selected using 2 mg/ml G418 (Gibco 11811031). 2Myc-WWOX (full-length WWOX cDNA cloned into a Myc-tagged pCMV vector (BD Clontech)^60^) and 3XFlag-DVL2 (wild-type DVL2 fused to a Flag-tag, Addgene, 24802) were used for the IP of WWOX and DVL2.

### RNA extraction, reverse transcription-PCR, and RT-PCR

Total RNA was prepared using Bio-Tri reagent (Biolab, Israel) as described by the manufacturer. One microgram of RNA was used to synthesize cDNA using a qScript cDNA Synthesis kit (QuantaBio, USA). qRT-PCR was performed using Power SYBR Green PCR Master Mix (Applied Biosystems, USA). All measurements were performed in triplicate and were standardized to the levels of UBC.

### mRNA profiling

Total RNAs from MDA-MB435S (EV, WWOX and WWOX WFPA) cells were extracted using a standard TRIzol^®^ method and were hybridized using Affymetrix GeneChip^®^ Human Genome U133 Plus 2.0 arrays. Raw data was deposited in GEO (accession number GSE142317). The quality assurance, calibration, data normalization and PCA for the Affymetrix cell format files were performed using Partek Genomics Suite 6.5 (Partek Genomics, St Louis, MO, USA). Genes with a four-fold expression difference and cutoff *FDR* < 0.05 (by the Beonferroni method) were designated as significant. In the heat map of the Hierarchical clustering “*red*” represents transcripts with increased expression, while “*blue*” represents transcripts with decreased expression.

Differentially expressed genes were analyzed using the Enricher dataset (http://amp.pharm.mssm.edu/Enrichr/) and the KEGG database. For pathway enrichment, genes were analyzed that were found to be changed in the WWOX group (downregulated) when compared to the other two groups (EV-Empty vector and WFPA-mutated WWOX).

### MiRNA/gene prediction and enrichment analysis

For downstream analysis, we considered all those miRNAs that were significantly (*P* value < 0.05) deregulated (|Linear FC| > 1.5) and expressed (with an expression greater than 50 counts in at least one condition) (Fig. 2a) from our previous work.^34^ G3 represents the WWOX group while G1 represents the EV and WFPA mutant groups. The heatmap was generated by using *heatmap.2* function from *gplots* R package. Spearman correlation was used as the distance in the hierarchical clustering for both rows and columns. For each filtered miRNA, we performed miRNA consensus target prediction analysis by employing miRWalk (v.2.0) tool.^61^ In particular, we analyzed the predicted targets with a minimum consensus of six out of twelve predictor tools and a minimum seed length of seven nucleotides (see Supplementary Fig. 1). The set of the predicted transcripts were compared with the list of significantly deregulated ones; more specifically, for each upregulated miRNA we took into consideration the list of downregulated genes, and vice versa, defining them as “*strong predicted*” targets. For the downstream analyses we filtered out both miR-151-5p and miR-320a-3p because they had no “*strong predicted*” targets. The filtered list of potential predicted targets of each miRNA was used as input in IPA^®^ functional/pathway enrichment analysis. During the enrichment analysis phase, we considered just the human experimental observations in both mammary gland and breast cancer cell lines.

### Luciferase assay

MDA-MB435S cells were co-transfected with 1 μg of p3′UTR-*SMAD3* or p3′UTR mut-*SMAD3* (deletion mutation), 0.1 μg of Renilla luciferase expression construct, and pRL-TK (Promega) using Lipofectamine 2000 (Invitrogen). Cells were harvested 24 h after transfection and were assayed with a Dual Luciferase Assay (Promega) according to the manufacturer’s instructions. Three independent experiments were performed, and each was performed in triplicate.

### WWOX immunoprecipitation (IP) and MS

MDA-MB435S (Ev, WWOX or WFPA overexpressing) cells were seeded in 14 cm plates. Twenty-four hours later whole cell extract was prepared using lysis buffer (NaCl 150 nM, Tris 50 nM, glycerol 5%–10%, NP-40 1%, PH = 7.4) supplemented with protease and phosphatase inhibitors. A preclearing step was performed using mouse anti-IgG (Invitrogen). For IP, a cocktail of mouse anti-WWOX monoclonal antibodies^60^ was used together with protein A/G plus agarose bead (Santa Cruz, Sc-2003); they were incubated together overnight while rotating at 4 °C. Beads were washed 3 times with washing buffer (NaCl 150 nM, Tris 50 nM, glycerol 5%, NP-40 0.05%, pH = 7.4). Proteins were then eluted using two elution buffers: Elution buffer 1—2 M urea, 50 mM Tris-HCl (pH 7.5), 1 mM DTT and 5 µg/ml trypsin; Elution buffer 2—2 M urea, 50 mM Tris-HCl (pH 7.5) and 5 mM Chloroacetamide (Supplementary material^62^). The eluted material was then injected into the MS machine (Q Exactive Mass Spectrometer, Thermo Scientific). Raw data was analyzed for putative hits in the WWOX (wild type) versus both EV (empty vector) and WFPA (mutated WWOX) groups. IP-MS was preformed using three biological replicates.

### WWOX-DVL2 interaction

To validate the WWOX-DVL2 interaction, 2 µg of 2Myc-WWOX and Flag-DVL2 were co-transfected in HEK293T cells. Twenty-four hours post-transfection, the cells were incubated with or without Wnt ligands for another 24 h. Whole cell extracts were isolated, pre-cleared with mouse anti-IgG (Invitrogen) and were subjected to IP overnight using anti-Myc (Santa Cruz, Sc-40), anti-IgG and anti-Flag (Sigma Aldrich, F1804) antibodies. Precipitates were washed, eluted and run on SDS-PAGE for immunoblotting with the indicated antibodies.

### Immunoblot analysis

Cells were lysed using lysis buffer containing 50 mM Tris (pH 7.5),150 mM NaCl, 10% glycerol, and 0.5% Nonidet P-40 that was supplemented with protease inhibitors. Western blotting was performed under standard conditions. Blots were repeated and quantified 2–3 times per experiment. Representative images of those repeated experiments are shown. Antibodies used were polyclonal WWOX,^60^ monoclonal GAPDH (Calbiochem, 6C5, CB1001), polyclonal anti-Fibronectin for immunoblotting (Sigma Aldrich, F3648), polyclonal anti-Fibronectin for immunohistochemistry (Abcam, Ab2413), anti-Myc (Santa Cruz, Sc-40), anti-Flag (Sigma Aldrich, F1804) and polyclonal Smad2/3 (Santa Cruz, sc-8332).

## Supplementary information

Supplemental Figures

Table S1

Table S2

Table S3

Table S4

Table S5

Table S6

Table S7
