# Supplementary material for: Pleiotropic tumor suppressor functions of WWOX antagonize metastasis
Source: Signal Transduct Target Ther. 2020 Apr 17;5:43. doi: 10.1038/s41392-020-0136-8 (PMC7162874; doi:10.1038/s41392-020-0136-8)
Supplement: Supplementary file 1 — Supplemental Figures [file 41392_2020_136_MOESM1_ESM.pdf]

Supplementary Materials for

Pleiotropic tumor suppressor functions of WWOX in antagonizing metastasis

Saleh Khawaled[1], Giovanni Nigita[2,*], Rosario Distefano[2,*], Sara Oster[1], Sung-Suk Suh[3], Yoav Smith[4], Abed Khalaileh[5], Yong Peng[6], Carlo M. Croce[2], Tamar Geiger[7], Victoria L. Seewaldt[8], Rami I. Aqeilan[1,2]

Correspondence to: ramiaq@mail.huji.ac.il

This PDF file includes:

Figures. S1 to S2
Captions for Tables S1 to S7

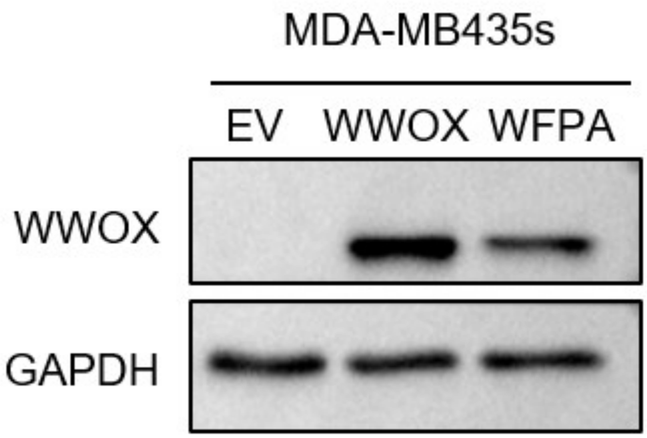

Figure. S1.

WWOX levels in manipulated MDA-MB435s cells.

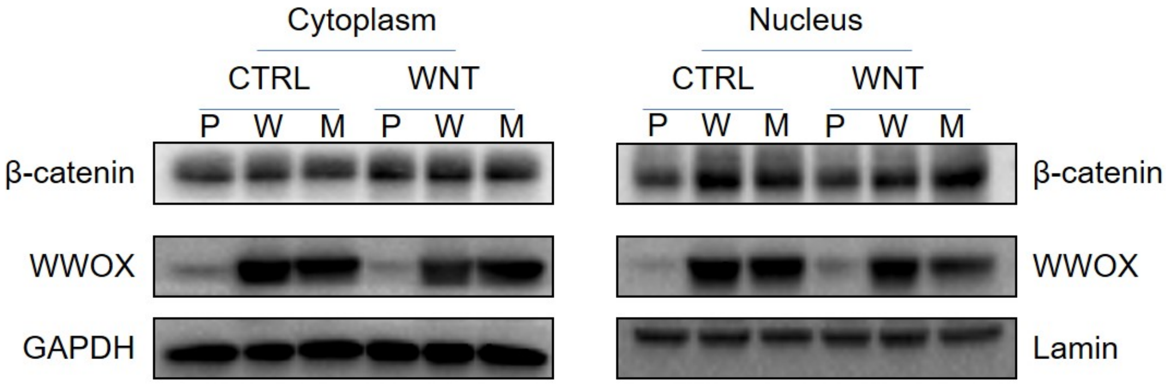

β-catenin/WWOX Ratio			
		Cytoplasm	Nucleus
CTRL	P	1	1
	W	0.3	0.26
	M	0.33	0.23
WNT	P	1	**1**
	W	0.33	**0.38**
	M	0.27	**0.64**

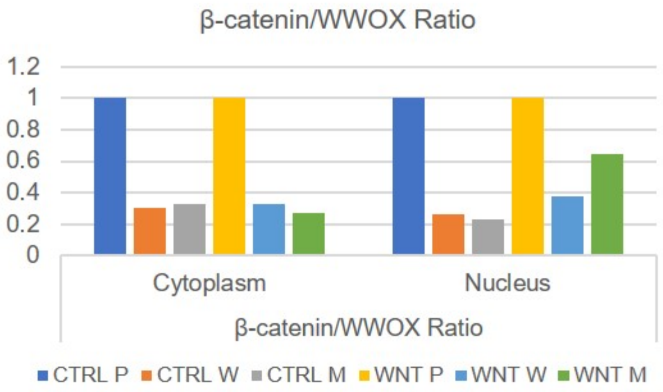

Figure. S2.

WWOX manipulation modulates nuclear β-catenin levels. This blot shows the levels of β-catenin in the cytoplasm and nucleus of MDA-MB-231 cells with or without WNT3A activation. Shown are the levels of β-catenin relative to the WWOX expression levels. Levels were normalized relative to unmanipulated parental cells. Higher levels of WT WWOX in the overexpressed cells result in lower levels of β-catenin in the nucleus, demonstrating a negative correlation between the two, and strengthening what is previously known about the relationship between WWOX and DVL2. Overexpression of mutant WWOX (WFPA) instead of WT shows a partial rescue of this.

Table S1.
mRNA expression profiling in empty-vector (EV), WWOX and WWOX-WFPA mutant – expressing in MDA-MB435s cells.

Table S2.
Enriched pathways from downregulated genes

Table S3.
Enriched pathways from upregulated genes

Table S4.
Predicted targets of miRNAs genechip experiments

Table S5.
mRNA expression of the predicted targets of the downregulated miRNAs

Table S6.
Pathway enrichment analysis for the “strong predicted” targets of the miRNAs

Table S7.
List of proteins that bind either WT WWOX, Mutant WFPA-WWOX or both in MDA-MB435s cells, obtained via Mass-spectrometry
